# Reliability Analysis of Flip-Chip Packaging GaN Chip with Nano-Silver Solder BUMP

**DOI:** 10.3390/mi14061245

**Published:** 2023-06-13

**Authors:** Lei Yan, Peisheng Liu, Pengpeng Xu, Lipeng Tan, Zhao Zhang

**Affiliations:** Jiangsu Key Laboratory of ASIC Design, School of Information Science and Technology, Nantong University, Nantong 226019, China

**Keywords:** flip-chip, solder bump, GaN power device, thermal reliability, thermal stress

## Abstract

Gallium nitride (GaN) power devices have many benefits, including high power density, small footprint, high operating voltage, and excellent power gain capability. However, in contrast to silicon carbide (SiC), its performance and reliability can be negatively impacted by its low thermal conductivity, which can cause overheating. Hence, it is necessary to provide a reliable and workable thermal management model. In this paper, a model of a flip-chip packing (FCP) GaN chip was established, and it was assigned to the Ag sinter paste structure. The different solder bumps and under bump metallurgy (UBM) were considered. The results indicated that the FCP GaN chip with underfill was a promising method because it not only reduced the size of the package model but also reduced thermal stress. When the chip was in operation, the thermal stress was about 79 MPa, only 38.77% of the Ag sinter paste structure, lower than any of the GaN chip packaging methods currently in use. Moreover, the thermal condition of the module often has little to do with the material of the UBM. Additionally, nano-silver was found to be the most suitable bump material for FCP GaN chip. Temperature shock experiments were also conducted with different UBM materials when nano-silver was used as bump. It was found that Al as UBM is a more reliable option.

## 1. Introduction

Power semiconductors play a crucial role in the effective distribution, utilization, and generation of energy in electric drive systems. In recent years, the emergence of the Wide Band Gap (WBG) semiconductor GaN has led to its gradual replacement of traditional semiconductor material silicon in numerous electronic devices and components. This new-generation semiconductor material is proving to be highly effective in improving the overall performance and energy efficiency of electric drive systems. In theory, wide bandgap technology enables power electronics to operate at temperatures exceeding 250 °C, as well as higher power densities and switching frequencies [1]. This technology has gained widespread use in various applications, including electric vehicles, radar, lighting, lasers, and power amplifiers [2,3,4,5,6].

Since WBG materials and their processing are expensive, minimizing the chip and package size of related devices is economically important for the commercialization of these devices. However, smaller sizes are often accompanied by increased self-heating, which affects the reliability of the chip. In addition, the assembled parts suffer from thermal stress due to switching frequently because of the different coefficients of thermal expansion (CTE). Presently, GaN chips are mainly packaged with direct bonded copper (DBC) or direct bonded aluminum (DBA) as the main structure that applied Ag sinter paste to improve their electrical, thermal, and mechanical properties and fatigue life [7,8]. Kim et al., improved the thermal stability by adding wolfram inside the Ag sinter paste, but this also increased the whole chip package size [9]. FCP is a new generation of packaging methods, often boasting the advantages of small size and high stability, suitable for GaN chip packaging. Daniel et al. used FC integration for thermal management for GaN and Ultra-Wide Bandgap (UWBG) power amplifiers and achieved a beneficial effect [10].

Nowadays, the power density of commercial GaN power chips is 4~8 W/mm^3^, so as to avoid damage to the chip due to the self-heating effect. However, this is only 20% of its theoretical limit. There have been experiments to reduce the self-heating effect of GaN power chips by placing them on SiC substrates, which have very high thermal conductivity [11]. In addition to that, the method of double-sided cooling is being used [10]. However, the thermal management of WBG power devices still has the following problems to be solved [12].

(I) High current density and electric field generates very high local heat flux resulting in irregular temperature distributions and local thermal runaway.
(II) High thermal resistance due to unreasonable device structure.

In this work, an FCP GaN module was compared with conventional packaging. The structure used SiC as the substrate and added underfill to enhance the stability of the structure. The relationship between temperature and stress was considered and different solder bumps were tried to investigate the effect of materials on the response to thermal stress in FCP. Through our simulation experiments, FCP was proved to be a feasible option that can be used for thermal management of WBG and UWBG power devices.

## 2. Models and Simulation

### 2.1. Modeling

**Figure 1:**
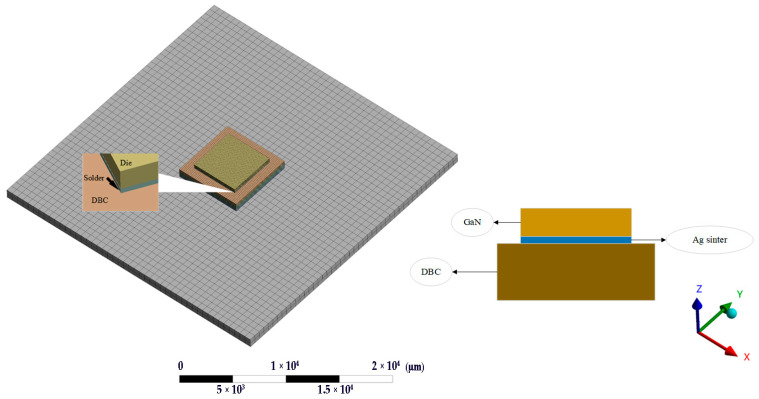
GaN/DBC module using Ag sinter paste.

**Figure 2:**
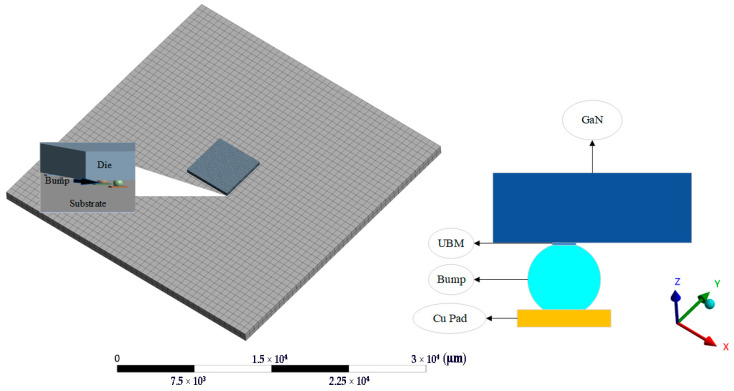
FCP GaN module and the structure of the solder bumps.

To contrast the reliability of FCP GaN and conventional packaging, two modules were established. The DBC package structure is shown in Figure 1, using Ag sinter paste to solder the GaN chip onto the DBC layer with a middle insulation layer of Si_3_N_4_. On the other hand, the FCP structure, shown in Figure 2, used a 5 × 5 array of solder bumps that had been simplified to place the chip upside down on the substrate. Both modules had chips and substrate of the same size.

The dimensions of each part of the module are shown in Table 1. Comparing Figure 1 and Figure 2, it can be seen that the FCP effectively reduces the whole module size. In addition, since the chip is inverted on the solder bumps, thermally conductive silicone grease can be painted on the back of the chip to enhance the heat dissipation capability. Furthermore, removing wire bonding is possible to achieve a higher density layout by redistributing layer (RDL) on the substrate without taking up space on the chip surface [13]. Hence, the distance between the chip and the substrate is shortened, the time delay of signal transmission is reduced, and the chip performance is improved [14].

The volume of the GaN/DBC module was 961.5 mm^3^ and the volume of the FCP GaN module was 910.04 mm^3^. Volume was reduced by 5.35%. Without counting the substrate, the volume of FCP GaN module was only 16.3% of that of the former.

### 2.2. Finite Element Method

Finite element method (FEM) analysis was used to verify the module. In the FEM module, when the switch is turned on and the chip is in operation, the heat generated by the chip transfers heat to other components and the environment via heat conduction, convection, radiation, etc. In the three-dimensional (3D) spatial coordinate system (x, y, z), the 3D transient heat transfer equation for heat conduction versus time is established by the first law of thermodynamics and Fourier’s heat conduction equation as Equation (1):

(1) $$\frac{k}{\rho c}(\frac{\partial^{2}T}{\partial x^{2}}+\frac{\partial^{2}T}{\partial y^{2}}+\frac{\partial^{2}T}{\partial z^{2}})=\frac{\partial T}{\partial t}$$

where k is the thermal conductivity (W/m·K), ρ is the density (kg/m^3^), c is the specific heat capacity (J/kg·K), T is the temperature (K), and t is the time (s). When the temperature of the whole module changes, thermal deformation occurs in the 3D spaces, which is mainly influenced by the support conditions, temperature difference, material properties, etc. The amount of thermal deformation Δ in the 3D direction can be expressed as Equation (2):

(2) $$\left\{ \begin{array}{l} \Delta_{x}=\alpha_{x}\int_{0}^{L_{x}}[T(x)-T_{0}]dx\\ \Delta_{y}=\alpha_{y}\int_{0}^{L_{y}}[T(y)-T_{0}]dy\\ \Delta_{z}=\alpha_{z}\int_{0}^{L_{z}}[T(z)-T_{0}]dz \end{array}\right.$$

where α is the CTE of the material (1/K) and T (x, y, z) is the temperature of the calculation point (K). The total deformation is shown in Equation (3).

(3) $$\Delta_{\mathrm{sum}}=\sqrt{\Delta_{x}{}^{2}+\Delta_{y}{}^{2}+\Delta_{z}{}^{2}}$$

Due to the different coefficients of the thermal expansion of materials, when there is a temperature difference, stresses are generated by squeezing each other inside the module, which is thermal stress. The equation of thermal stress can be calculated by the coefficient of thermal expansion, the temperature difference, and the mechanical properties of the component material. The formula for thermal stress is shown in Equation (4):

(4) σ=α⋅E⋅ΔT

where σ means the thermal stress of the chip (GPa); E is the elastic modulus of the chip material (GPa); and ΔT means the temperature difference between different parts inside the module (K). The above formulae show that the thermal stress of the model is related to the coefficient of thermal expansion, the temperature difference, and the elastic modulus of the material. When the temperature difference inside the module is large, the thermal stress will increase, which may lead to chip failure or damage. Before conducting the FEM analysis, the material of each component of the module is defined, and Table 2 shows the material properties of each component [15].

### 2.3. Simulation

**Figure 3:**
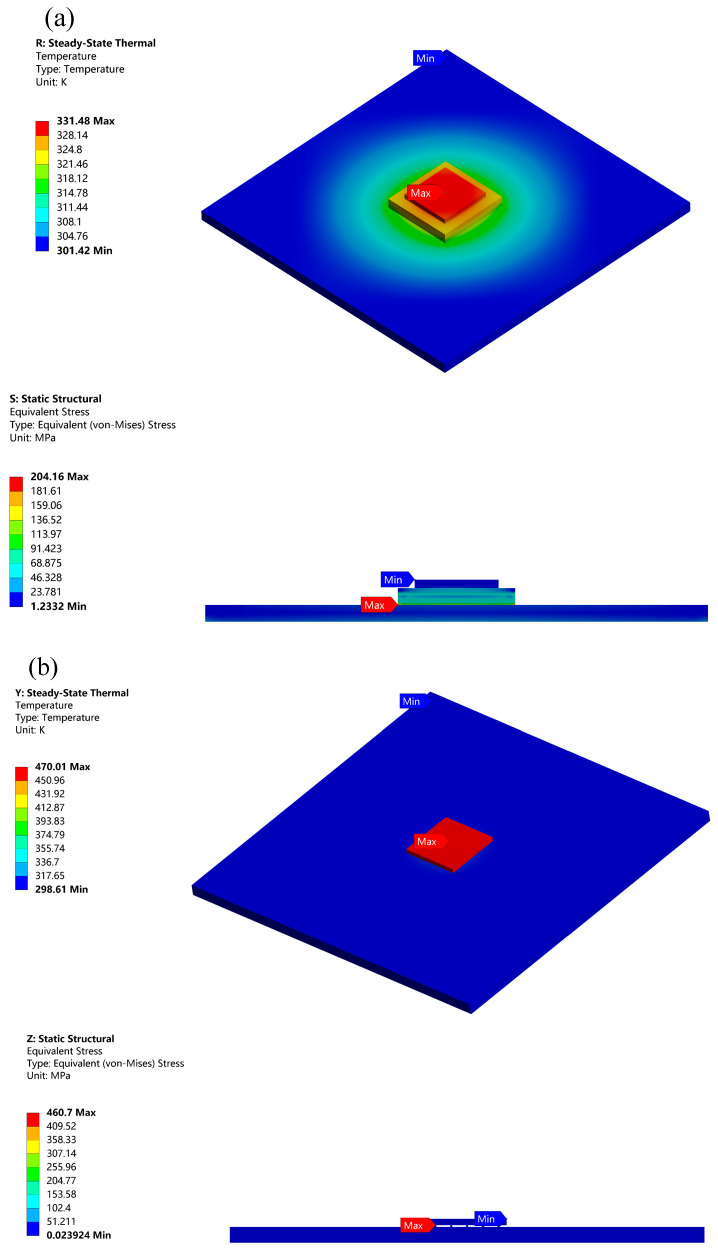
Thermal stress analysis of (**a**) GaN/DBC package (**b**) FCP.

FEM analysis was conducted using ANSYS WorkBench platform at room temperature (295.15 K) and the findings are presented in Figure 3. The simulation indicated that, despite its smaller size, the FCP encountered a serious heat dissipation issue at the same power density, reaching a maximum temperature of 470.01 K, which was considerably higher than that of the DBC package structure. This was primarily due to the inadequate heat conduction between the chip and the substrate, which was solely facilitated by the 5 × 5 Bump array. To address this problem, underfill was added between the chip and the substrate [16]. By adding underfill, it not only optimized the heat conduction of the FCP and better protected the bumps, but also freed the bumps from electrostatic interference and increased the stability of the bumps [17,18].

**Figure 4:**
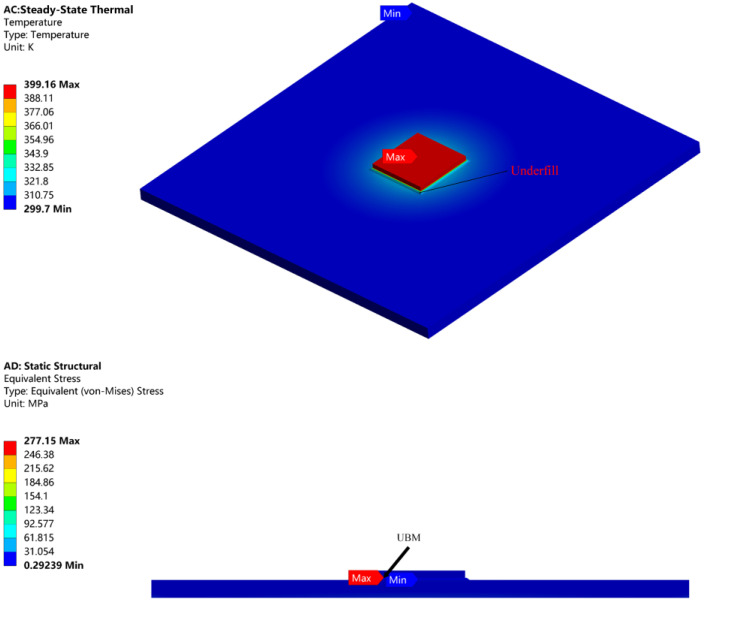
Thermal stress analysis of FCP with underfill.

**Figure 5:**
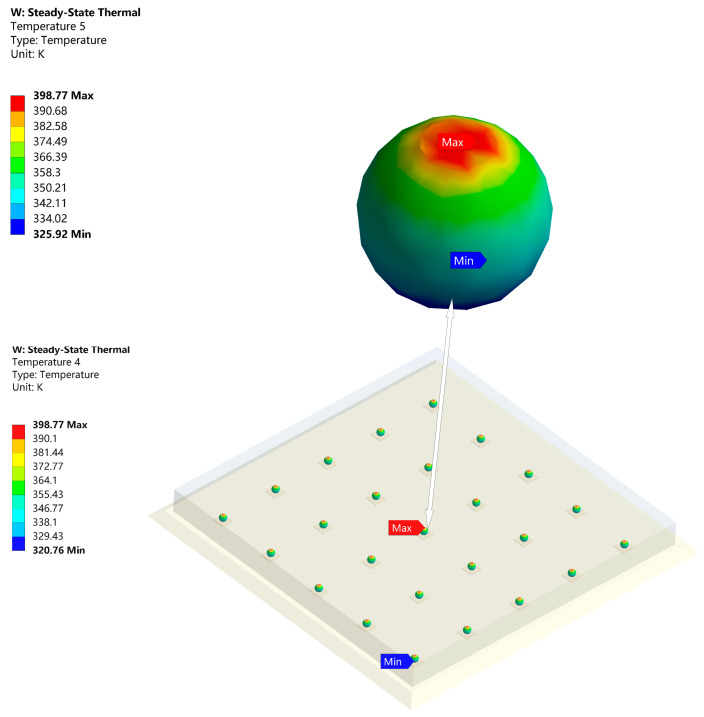
Temperature distribution of solder bumps (SnPb).

Figure 4 shows the temperature and thermal stress after adding underfill. The maximum temperature was reduced to 399.16 K. Clearly, the underfill plays a significant role in heat dissipating and supporting [18,19,20,21]. In contrast to Figure 3, we known that the reliability of the FCP was worse compared to the Ag sintered package. The maximum temperature of the FCP GaN chip was 67.68 K higher than that of DBC, while the temperature of the substrate was lower. In Figure 5, the temperature distribution between the top and bottom of the solder bumps reached 72.85 K. This is probably because the k of the traditional solder bump material is poor, which caused the large temperature difference.

Due to the reduced size, the heat dissipation of the module is relatively affected, and the UBM layer is subjected to the maximum stress, which seriously affects the reliability of the FCP. Optimization of FCP GaN chip is required.

Pairing Equations (1) and (4), it can be inferred that the reliability problem of FCP is related to the properties of materials [10,12,22,23,24,25,26]. For finding the most suitable material, Taguchi’s experimental method was used [27]. Different combinations of bump and UBM materials are employed to enhance the thermal reliability of the FCP GaN chip. Table 3 shows the properties of the different materials, and there are 12 groups of experiments to find the most suitable bump and UBM material. The experimental results are shown in Table 4.

**Figure 6:**
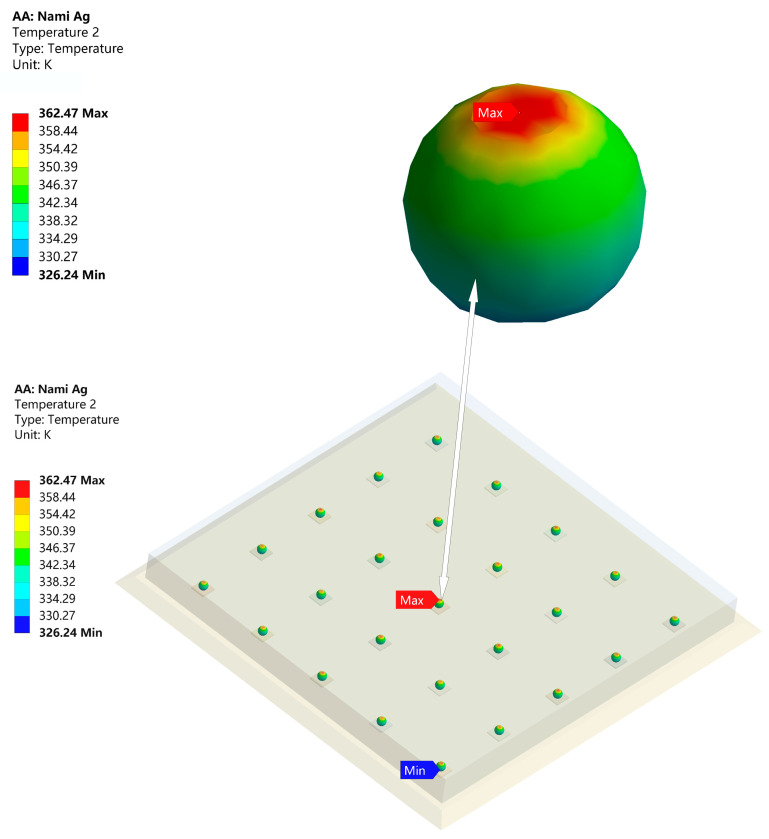
Temperature distribution of solder bumps (Nano-Ag).

From Table 4, it is clear that when bump is determined, the material of UBM has little effect on the whole module heat conduction. This is logical since the size of UBM is much smaller than bump and underfill. Its impact on the module’s heat conduction was negligible. Figure 6 shows the temperature distribution of the solder bumps with the best optimization results. The maximum/minimum temperature difference is 36.23 K. Matching with Figure 5, its thermal resistance is reduced by 50%. As a result, nano-silver is the optimal material as a bump for FCP GaN chip, with less thermal stress when the UBM is chosen as Al and better thermal conduction when the UBM is chosen as Cu. Both nano-silver’s thermal conductivity and mechanical properties are superior to other materials [28,29,30,31]. Since UBM tends to be subjected to the maximum thermal stress, temperature shock experiments were conducted on FCP GaN chip of UBM(Al) and UBM(Cu) to evaluate the reliability of the modules [32].

## 3. Temperature Shock Experiments

Considering the relationship between the temperature and stress–strain of the bumps, the Anand model of nano-silver was applied [33]. It can produce a more accurate analysis of the resistance to deformation under temperature shock. Table 5 shows the parameters of the Anand model of nano-silver.

**Figure 7:**
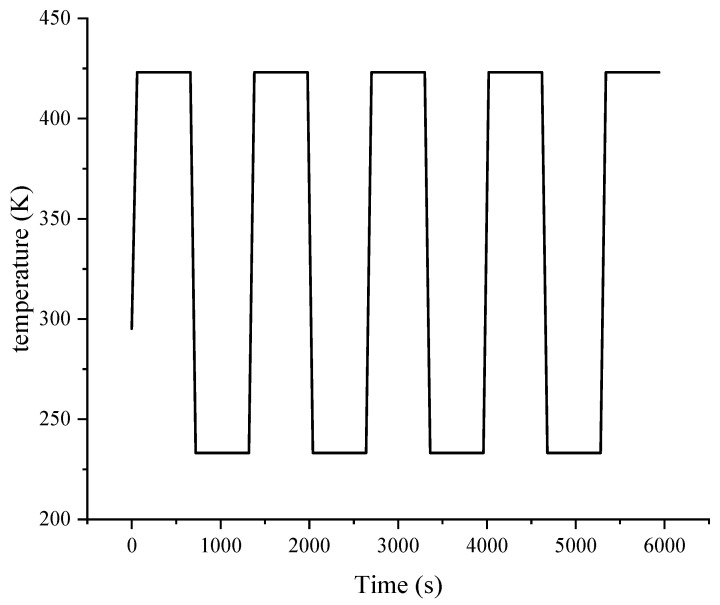
Conditions of temperature shock.

The range of temperature shock is −40–150 °C (233 K–423 K), the high and low temperatures lasting for 600 s with 60 s as the period of transition. Temperature shock damage increases with the number of cycles, but generally tends to stabilize the fatigue damage change after four cycles. Figure 7 shows the temperature shock conditions. High and low temperatures lasted four cycles for a total of 6000 s.

**Figure 8:**
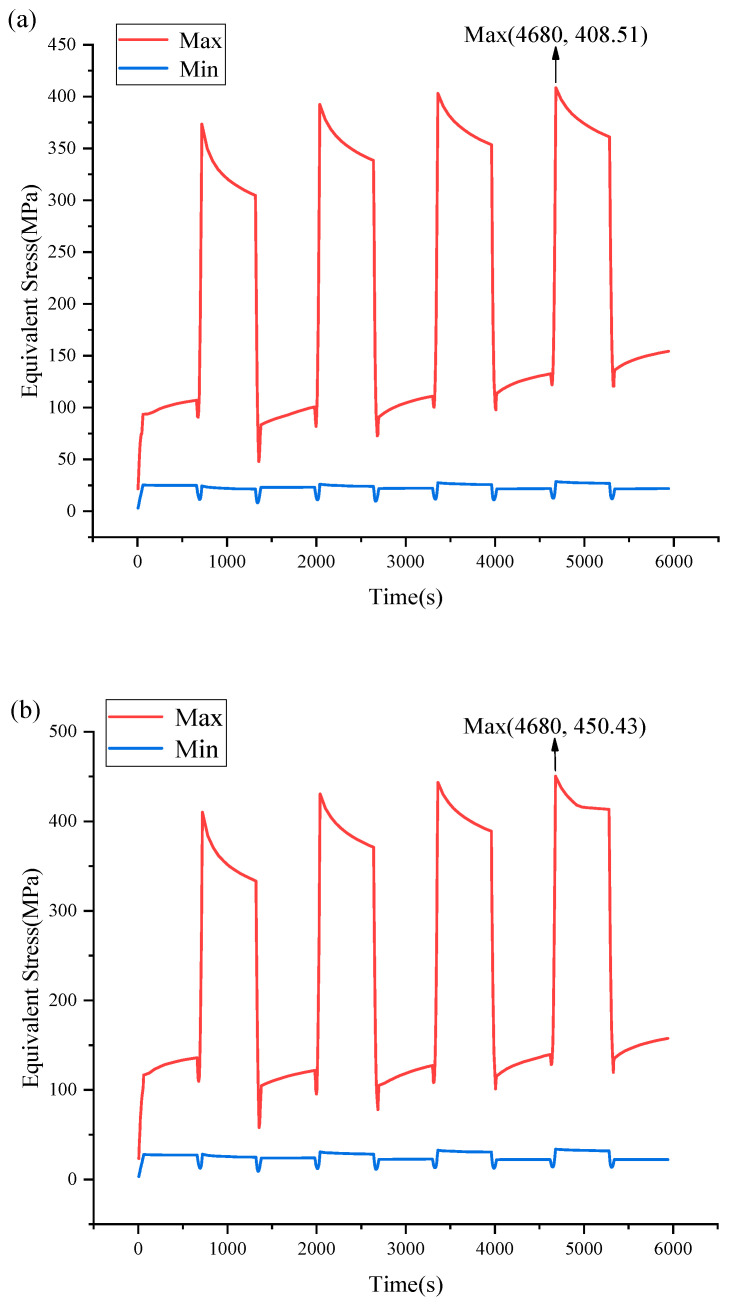
Equivalent stress evolution diagram under temperature shock of (**a**) UBM(Al)/(**b**) UBM(Cu).

Figure 8 shows the results of the temperature shock experiments, (a) for UBM(Al) and (b) for UBM(Cu). The maximum Von-mises stress was 408.21 MPa for UBM(Al) and 450.43 MPa for UBM(Cu). Both were obtained at 4680 s. It is evident from Figure 8 that, after the third temperature shock, the maximum Von-mises stress hardly changed again.

In summary, the FCP GaN chip is workable at the same power density. It supports a smaller size although heat conduction needs to be continually improved. Considering that a heat sink can be placed directly on the back of the chip, it is more suitable in high power and high temperature conditions [34,35]. It also offers higher resistance to electrostatic interference and deformation due to the protection of the underfill.

## 4. Conclusions

By comparing the thermal reliability of conventional DBC package structure and FCP structure of GaN chips at the same power density, the advantages and disadvantages of FCP GaN chips were displayed. FCP can reduce the size of the whole module, and without wire bonding it avoids the chip failure problem caused by bonding wire failure. Moreover, underfill was added to avoid signal distortion caused by electrostatic crosstalk. The thermal stress of FCP was greatly reduced. Through comparison of simulations, it is easy to see that the preferred solder bump material for FCP GaN chip is nano-sliver, which has a very strong heat dissipation capacity. Furthermore, its coefficient of thermal expansion matches GaN well. Maximum equivalent stress was also lower than the DBC package structure, only 79.184 MPa; UBM is often subjected to the maximum stress. Through temperature shock experiments, Al as UBM material was found to be a good choice.

However, the FCP GaN chip still faces many challenges. Currently, the power density of GaN power chip is far less than the theoretical power density value of 42 W/mm^3^. Within the study of power density, FCP GaN chip heat conduction capacity is still inferior to the DBC double-sided cooling package structure. Optimizations are still needed to improve the thermal reliability of the FCP structure. The currently viable options are as follows:

(I) Using mixed materials with higher k instead of epoxy resin as underfill, allowing heat to pass between the chip and the substrate more quickly.
(II) Reducing the temperature of high-power devices with dual-small-outline (DSO) packages (from Infineon). DSO allows for junction-side cooling, which is suitable for semiconductor materials with low k and can solve the problem of irregular thermal distribution in lateral GaN power devices.
(III) Adopting a PCB embedding method. This method has achieved good heat conduction results in both individual GaN devices and GaN integrated circuits.

## Figures and Tables

**Table 1:** Module size parameters.

Model	Size (μm)	Thickness (μm)	Diameter (μm)
Substrate	30,000 × 30,000	1000	
Die	5000 × 5000	400	
DBC	7000 × 7000	300	
	7000 × 7000	400	
	7000 × 7000	300	
Ag sinter	5000 × 5000	100	
Cu pad	200 × 200	20	
Bump		95	120
UBM		5	55

**Table 2:** Material properties.

Material	Density (kg/m^3^)	CTE (/K)	Thermal Conductivity (W/m·K)	Young’s Modulus (GPa)	Poisson’s Ratio	Specific Heat (J/kg·K)
GaN	6100	5.6 × 10^−6^	110	211	0.17	412
Ag	6294	3.25 × 10^−6^	430	12.9	0.1	240
Cu	8950	1.64 × 10^−5^	393	110	0.34	385
Si_3_N_4_	3200	2.6 × 10^−6^	100	300	0.28	700
UBM	8900	1.34 × 10^−5^	91	200	0.31	443.8
Substrate	3210	5.1 × 10^−6^	150	400	0.142	710

**Table 3:** Properties of bump and UBM.

Part	Material	CTE (/K)	Thermal Conductivity (W/m·K)	Young’s Modulus (GPa)	Poisson’s Ratio
Bump	SnPb	2.4 × 10^−5^	50	19.7	0.4
	SAC305	2.3 × 10^−5^	57.26	26.2	0.35
	Nano-silver	1.96 × 10^−5^	238	80	0.37
	Au	1.44 × 10^−5^	318	79	0.44
UBM	Ni	1.34 × 10^−5^	91	200	0.31
	Al	2.3 × 10^−5^	240	69	0.33
	Cu	As shown in Figure 2			

**Table 4:** Results of Taguchi’s experimental method.

Combination	Maximum Temperature (K)	Maximum Von-Mises Stress (MPa)
SnPb + Ni	399.16	277.15
SnPb + Al	399.12	197.61
SnPb + Cu	399.09	232.49
SAC305 + Ni	408.06	374.82
SAC305 + Al	408.07	266.3
SAC305 + Cu	408.08	320.84
Nano-silver + Ni	368.79	97.837
Nano-silver + Al	368.67	79.148
Nano-silver + Cu	368.59	89.112
Au + Ni	377.31	575.21
Au + Al	376.79	529.35
Au + Cu	377.05	391.26

**Table 5:** Anand’s model parameters of nano-silver.

*A* (s^−1^)	*Q*/*R* (1/K)	*m*	*n*	ξ	*s’* (MPa)	*h*_0_ (MPa)	*s*_0_ (MPa)	α
9.81	5709	0.6572	0.00326	11	67.389	15,800	2.768	1

## Data Availability

Not applicable.
